# Moral hypocrisy in economic games—how prosocial behavior is shaped by social expectations

**DOI:** 10.3389/fpsyg.2014.00897

**Published:** 2014-08-14

**Authors:** Lucius Caviola, Nadira Faulmüller

**Affiliations:** ^1^Department of Experimental Psychology, University of OxfordOxford, UK; ^2^Oxford Martin School, University of OxfordOxford, UK

**Keywords:** prosocial behavior, fairness, expectations, social perception, economic games, anonymity, moral hypocrisy

## Introduction

Experimental research in behavioral economics has revealed a high degree of prosocial tendencies in human interactions. These results have been interpreted as suggesting a necessary shift from a model of *selfish preferences* toward *social preferences*—the assumption that people intrinsically are concerned about others' well-being (e.g., Fehr and Schmidt, 1999; Bolton and Ockenfels, 2000). In this article, we first introduce research on prosocial behavior from an economics perspective. We then review several studies that invalidate this *social preferences hypothesis* by demonstrating that prosocial behavior is reduced when behavior is not observable by relevant others and expectations to behave prosocially are lowered. We argue that people primarily pursue self-interest as long as they can retain the appearance of being fair to others and even themselves—an interpretation referred to as *moral hypocrisy* (Batson, 2011). Further, we highlight implications for research of this assumption.

In behavioral economics, prosocial behavior toward strangers has been examined using the *Dictator Game* (DG) in which two strangers anonymously interact with each other, typically via computers so that they cannot see each other. Classic versions of this game are played under perfect information, meaning that both players are informed about the rules and the possible actions each player may take. One person is randomly assigned the role of the dictator and the other person the role of the recipient. Dictators get an initial $10 and are given the opportunity to decide how much money they want to hand over to the recipient. Recipients, in contrast, begin the game without any initial endowment and cannot influence their final payoff. Clearly, a selfish dictator would keep everything. However, a meta-analysis by Engel (2011) covering 41,433 single DGs shows that on average, people give about 28.35% of their endowment to the other person and 17% of the dictators give half of it. Henrich et al. (2004) confirmed these findings in cross-cultural studies. Similar cooperative tendencies are found in other economic games like the *Ultimatum Game*, the Third *Party Punishment Game* and the *Public Good Game* (Camerer, 2003). Behavioral economists have tried to explain this kind of prosocial behavior by assuming that people have *social preferences*. Some of their models suggest that people are intrinsically motivated to reach fair distributive outcomes (cf. Fehr and Schmidt, 2006). Fehr and Schmidt (1999), for instance, assume that people are *inequity averse* and therefore try to minimize the difference between distributional outcomes. Even though these models are successful in explaining a large amount of data, several studies seem to question their underlying assumption. These studies add a more psychological angle by subtly modifying economic games in a way that manipulate participants' beliefs about what behavior others might expect from them.

## Meeting other participants' expectations

Dana et al. (2006) introduced a modified version of the DG, which allowed dictators to eliminate the recipient's expectations. As in the classic DG, dictators could decide how to split their $10. After they have made their putative decision, but right before its implementation, the experimenter intervened offering the following option: either dictators could go ahead with their decision and divide the amount of money as intended, or they could choose to pay $1 in order to exit the game. In this case, the recipient would never be told that the game happened in the first place. Hence, dictators who exited only received $9 while the recipient received nothing. Assuming people were truly motivated to ensure fair distributive outcomes, we would assume that participants would stick to their primary decision and split the money fairly. However, in two experiments 28% and 43% of the dictators chose the exit option independently of their primary decision. Some of the participants initially decided to split fairly but as soon as they knew the recipient was not expecting anything, they behaved selfishly. An interpretation could be that people were willing to pay money to make sure others would not find out. This is in sharp contrast to what models of money-maximizing preferences (keeping the full amount) or social preferences (splitting fairly) would predict.

Similarly, in another study, participants were playing a modified version of the *Ultimatum Game* involving asymmetric information regarding the amount of money to be distributed (Overgaauw et al., 2012; for a similar study see Kagel et al., 1996). In the classic Ultimatum Game proposers can make an offer of how to divide a sum of money. If the recipients accept, the money is split accordingly. If they reject, neither proposer nor recipient receive anything. In this modified version, however, the proposer was able to see the complete amount of money, whereas the recipient saw only a part of it (e.g., the proposer saw all 10 coins, the recipient only 8). The proposer was aware of this asymmetry. The results revealed that proposers gave less money when information was hidden from the other person. The more information was hidden, the lower the amount of money offered. Intriguingly, children at age eight already show this kind of strategic—and clearly selfish—behavior (Overgaauw et al., 2012).

Prosocial behavior also depends on the available set of options. List (2007) conducted a series of DG experiments that introduced the possibility of taking money away from the recipient. Dictators were endowed with $10 and receivers with $5. In the baseline condition, dictators were given the option to transfer between $0 and $5 to the receiver. 71% of the participants gave a positive amount, and on average participants gave $1.33, which reflects the expected findings from the previous DG literature. Dictators in the second condition, however, were given the option to transfer between $0 and $5 or alternatively take $1 away from the receiver. Here only 35% gave a positive amount, and on average participants gave $0.33. Dictators in the third condition were given a symmetrical set of options: they could either take up to $5 or give up to $5. Here, only 5% gave a positive amount, and on average participants took $2.48 away. These findings indicate that the available set of options can shift people's expectations about which actions are socially acceptable: if one has the opportunity to give up to a certain amount of money to another person, a fair split is expected. But if one has the opportunity to either give or take money, giving nothing or even taking a small amount away seems less bad because taking away everything would have been even worse.

One interpretation of these studies is that people behave prosocially only if, by doing so, they are able to meet the recipient's expectations. Notably, there seems to be a crucial difference for people between knowing that their identity is concealed (anonymity) and knowing that *someone* expects them to behave in a certain way. Even if people are interacting completely anonymously with a stranger who cannot see them and doesn't know their names, they might still feel the pressure of his or her expectation. But when people are able to evade social expectations, prosocial behavior is reduced.

## Meeting the experimenter's expectations

Participants' behavior can also be influenced by the presence of the experimenter. To counteract this observer-expectancy effect, Hoffman et al. (1994) conducted a double-blind Ultimatum Game in which the dictator's decisions were known neither to the experimenter nor the recipient. The authors describe a dramatic decrease of cooperation in double-blind conditions and conclude: “Other-regarding behavior is primarily an expectations phenomenon, rather than the result of an autonomous private preference for equity.” (p. 1). However, conventional double-blind settings might still not fully convince participants that they are not being observed. Hence, Franzen and Pointner (2012) applied the “randomized response technique” (Warner, 1965), which should guarantee perfect anonymity to participants. The technique involves two random devices that allow participants to give answers that are completely concealed from the experimenter. As suspected, dictators behaved even more selfishly in this condition than the standard double-blind condition. Thus, the more convincing the concealment of their actions, the less people feel pressured by the expectations of the experimenter. Furthermore, people's prosocial behavior might be influenced by an even subtler form of pressure than by other people's expectations, namely by their own expectations.

## Meeting one's own expectations

Prosocial behavior can be reduced when the connection between choices and outcomes is obfuscated. In another modified version of the DG, dictators could choose between two options: they would receive $5 for choosing option A and $6 for choosing option B (Dana et al., 2005). However, at this point they didn't know how much the recipient would get, which was either $5 or $1. If dictators wanted, they could decide, at no cost to themselves, to acquire information about the recipient's true payoffs before choosing an option. Participants were told that the recipient would not be informed about this decision. However, in this experiment only half of the dictators decided to acquire this information while the other half preferred to remain ignorant and chose the option that was in their best self-interest. Maybe dictators were reluctant to acquire the payoff information in order to evade the possibility of having to meet their own expectation of behaving fairly. By leaving the relationship between their decision and the recipient's payoff uncertain, they were able to preserve the illusion of behaving fairly.

## Implications

In conclusion, these studies suggest that many people are not interested in fair outcomes *per se*. Models of *social preferences* that only take into account the final distributional payoffs are not able to explain the decline of prosocial behavior in these studies. Instead, preference models should also take into account psychological factors such as social expectations. More specifically, it seems that many people do not behave prosocially because they intrinsically want to, but because they are driven by the expectations of other participants, experimenters, and even themselves. Perhaps, people are trying to meet social expectations in order to avoid negative feelings such as shame or guilt as a result of appearing unfair (Charness and Dufwenberg, 2006). This might hold true even if in fact people are just retaining the illusion of not being unfair.

This interpretation is in line with findings and theories from social psychological research. Daniel Batson and colleagues have argued that true moral motivation is less prevalent than often assumed (Batson et al., 1997, 1999; Batson, 2011). Instead, they theorize, people are motivated to *appear* moral without necessarily acting according to the moral standard—a phenomenon called *moral hypocrisy*. They empirically demonstrated, for example, that people assigned other participants unfairly to tasks when personal costs were at stake and that allegedly fair coin flips were interpreted self-servingly (Batson et al., 1997). Moreover, people behaved more fairly if they had to observe themselves in a mirror, presumably because by doing so the discrepancy between their own actions and the moral standard would be too salient (Batson et al., 1999). Batson and colleagues assume that people are deceiving others and themselves to believe that their actions are moral by either misperceiving their actions as moral or by failing to compare their actions to the moral standard.

It seems that the theory of moral hypocrisy as well as other influential work in the tradition of social psychology, which discovered conditions for prosociality decades ago (e.g., Darley and Latané, 1968; Batson et al., 1981; Levine et al., 2005), have only in parts been integrated in behavioral economics. This even though these theories can account for the finding that people are evading social expectations in order not to have to engage in costly prosocial behavior. It could be considered a failure of collaboration that these theories and findings have not found their way into behavioral economics earlier. One explanation could be the different methodology between the two fields. The studies presented in this article stayed in the framework and methodology of behavioral economics, and therefore, have direct implications for findings from that tradition. We hope that they will help to bridge the gap between economic and social psychological theories of human prosocial behavior. Furthermore, we urge researchers from both fields to ensure a closer inter-disciplinary collaboration.

### Conflict of interest statement

The authors declare that the research was conducted in the absence of any commercial or financial relationships that could be construed as a potential conflict of interest.
